# Fabrication of Polydopamine‐Coated High‐Entropy MXene Nanosheets for Targeted Photothermal Anticancer Therapy

**DOI:** 10.1002/advs.202410537

**Published:** 2024-12-24

**Authors:** Qingshuang Zou, Ailin Qiu, Yan He, Evelyn Y. Xue, Lujie Wang, Gun Yang, Yao Shen, Dixian Luo, Quan Liu, Dennis K. P. Ng

**Affiliations:** ^1^ Department of Chemistry The Chinese University of Hong Kong Shatin, N.T. Hong Kong 999077 China; ^2^ Department of Laboratory Medicine Huazhong University of Science and Technology Union Shenzhen Hospital (Nanshan Hospital) Shenzhen University Shenzhen 518052 China; ^3^ Institute of Pharmacy and Pharmacology School of Pharmaceutical Science Hengyang Medical School University of South China Hengyang Hunan 421001 China; ^4^ Department of Biomedical Engineering The Chinese University of Hong Kong Shatin, N.T. Hong Kong 999077 China

**Keywords:** cancer therapy, epidermal growth factor receptor, high‐entropy MXenes, photothermal therapy, polydopamine

## Abstract

Transition metal carbides, nitrides, and carbonitrides (MXenes) have emerged as a promising class of 2D materials that can be used for various applications. Recently, a new form of high‐entropy MXenes has been reported, which contains an increased number of elemental species that can increase the configurational entropy and reduce the Gibbs free energy. The unique structure and composition lead to a range of intriguing and tunable characteristics. Herein, the fabrication of high‐entropy MXene TiVNbMoC_3_T*_x_* (T = surface terminations) with a layer of polydopamine is reported, followed by immobilization of a phthalocyanine‐based fluorophore for imaging and the peptide sequence QRHKPREGGGSC for targeting the epidermal growth factor receptor (EGFR) overexpressed in cancer cells. The resulting nanocomposite exhibits high biocompatibility and superior photothermal property. Upon laser irradiation at 808 nm, the light‐to‐heat conversion efficiency is up to 56.1%, which is significantly higher than that of conventional 2D materials. In vitro studies show that these nanosheets could be internalized selectively into EGFR‐positive cancer cells and effectively eliminate these cells mainly through photothermal‐induced apoptosis. Using 4T1 tumor‐bearing mice as an animal model, the nanosheets could accumulate at the tumor and effectively eradicate the tumor upon laser irradiation without causing noticeable adverse effects to the mice.

## Graphical abstract

The high‐entropy MXene TiVNbMoC_3_T*_x_* (T = surface terminations) is coated with a layer of polydopamine and further conjugated with a fluorescent probe and a tumor‐targeting peptide on the surface. The resulting nanocomposite exhibits high biocompatibility and superior photothermal property for effective elimination of target cancer cells and tumors in mice.

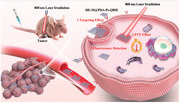

## 1 Introduction

MXenes refer to a family of 2D transition metal carbides, nitrides, and carbonitrides, which were first discovered in 2011.^[^ ^1^ ^]^ Since then, they have received considerable attention and experienced rapid development.^[^ ^2^ ^]^ With diverse compositions and structures, these materials exhibit extraordinary characteristics, such as high metallic electrical conductivity, strong in‐plane mechanical stiffness, tunable surface chemistry, strong absorption of electromagnetic radiation, and high hydrophilicity. Owing to these superior properties, MXenes have emerged as promising materials for a wide range of applications in energy storage and conversion,^[^ ^3^ ^]^ sequestration of environmental pollutants,^[^ ^4^ ^]^ sensors,^[^ ^5^ ^]^ etc. Inspired by the intriguing high‐entropy alloys,^[^ ^6^ ^]^ high‐entropy MXenes (HE‐Ms) were first reported in 2021.^[^ ^7^ ^]^ By introducing additional elemental species, the configurational entropy of MXenes can be increased, which can lower the Gibbs free energy and reshape the structural ordering, phase stability, and local electronic states of the materials.^[^ ^8^ ^]^ The highly dispersed constituent metals in the distorted lattice also result in exposed active sites, which can enhance their catalytic performance. Despite these advantageous features and our enhanced understanding on their structure‐property relationships, the development of HE‐Ms is still in its infancy. While a first‐principles theoretical study has been conducted to determine the synthesizability of HE‐Ms,^[^ ^9^ ^]^ only a few types of these 2D materials have been synthesized and investigated experimentally so far. To the best of our knowledge, their applications have been restricted to energy storage and catalysis,^[^ ^10^ ^]^ photodetection in optoelectronic devices,^[^ ^11^ ^]^ and antibacterial infection through photo‐ and sonoinduced therapies.^[^ ^12^ ^]^

To extend the application of HE‐Ms further in the biomedical arena, we report herein a tailor‐fabricated nanosystem for antitumor purpose, utilizing the superior photothermal property of these materials as the therapeutic tool. Efficient photothermal conversion is a common characteristic of high‐entropy materials.^[^ ^13^ ^]^ Although the underlying mechanisms have not been fully elucidated, it is believed that the process is predominantly governed by the localized surface plasmon resonance effect and interband transitions, which can be enhanced by the diverse composition of these materials. HE‐Ms are no exception, and this property renders them potentially useful for photothermal therapy (PTT), which has emerged as a promising treatment modality for cancer.^[^ ^14^ ^]^ It involves the excitation of a photosensitive material by near‐infrared light to induce local hyperthermia for tumor elimination. Compared with the traditional anticancer therapies, it exhibits minimal invasiveness, low systemic toxicity, and high spatiotemporal selectivity. While various 2D materials, such as graphene oxide, black phosphorus, and layered double hydroxide nanosheets have been used as photosensitizers for PTT,^[^ ^15^ ^]^ HE‐Ms have been little studied for this application, and their photothermal effect has only been applied for elimination of bacteria and their biofilms,^[^ ^12^ ^]^ not for tumor eradication, though a few other high‐entropy nanomaterials have been reported for antitumoral PTT very recently.^[^ ^13^, ^16^ ^]^ This background provided the impetus for this study, which aimed to take advantage of the superior photothermal property of these intriguing 2D materials to extend their therapeutic application against this deadly disease.

**Figure 1:**
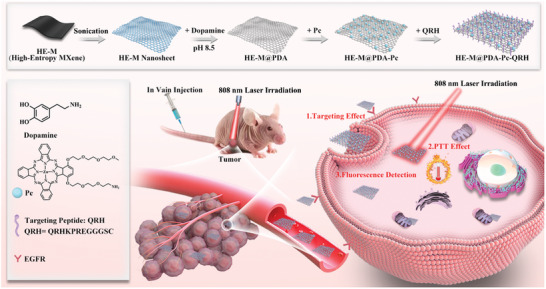
Synthetic route and antitumor mechanism of HE‐M@PDA‐Pc‐QRH.

The nanosystem contained HE‐M nanosheets of the formula TiVNbMoC_3_T*_x_* (T = surface terminations) as the core. With an equimolar proportion of four constituent transition metals, this material exhibits high configurational entropy, enabling the formation as a phase‐pure HE‐M.^[^ ^7^ ^]^ To enhance the biocompatibility, the nanosheets were coated with a layer of polydopamine (PDA), which is a highly versatile material for drug delivery.^[^ ^17^ ^]^ Owing to the presence of various reactive functionalities, such as amine, imine, and catechol on the surface, this coating can also facilitate the conjugation of various theranostic components. To enable visualization of the HE‐M nanosheets through fluorescence imaging, a zinc(II) phthalocyanine‐based fluorophore (labeled as Pc) was first immobilized on the surface.^[^ ^18^ ^]^ It was then followed by conjugation with the peptide sequence QRHKPREGGGSC (labeled as QRH), in which the heptapeptide QRHKPRE had been found to exhibit high and selective affinity toward the epidermal growth factor receptor (EGFR) overexpressed on the membrane of a wide range of human cancer cells.^[^ ^19^ ^]^ It was expected that the resulting nanocomposite HE‐M@PDA‐Pc‐QRH could be internalized selectively into EGFR‐positive cancer cells, and upon irradiation by near‐infrared light, the HE‐M core would induce local hyperthermia at the tumor site for effectively elimination of the tumor (**Figure** **1**).

## 2 Results and Discussion

### 2.1 Fabrication and Characterization

HE‐M nanosheets were first prepared by aqueous‐phase exfoliation and differential centrifugation of a commercial sample of TiVNbMoC_3_T*_x_* (Figure S1, Supporting Information). At a rotational speed of 9000–12 000 revolutions per minute (rpm), the batch of nanosheets collected showed a hydrodynamic diameter of 123.7 ± 9.1 nm as determined by dynamic light scattering (DLS). Increasing the rotational speed to 12 000–14 500 rpm led to the isolation of smaller nanosheets (94.8 ± 3.6 nm), which were more appropriate for biological studies. Although further increasing the rotational speed to >14 500 rpm enabled the collection of even smaller nanosheets (87.3 ± 5.5 nm), the concentration was very low. Hence, the second batch of sample was selected for further modification.

These HE‐M nanosheets (100 µg mL^−1^) were then mixed with dopamine (100 µg mL^−1^) in Tris‐HCI buffer pH 8.5 (10 mm), followed by sonication at room temperature for 3 h. Under these conditions, dopamine underwent self‐polymerization to form a coating on the HE‐M nanosheets. The resulting HE‐M@PDA (100 µg mL^−1^) was then treated with our previously reported amino phthalocyanine Pc (Figure 1)^[^ ^20^ ^]^ (5 µm) in deionized water with 0.5% (v/v) Tween 20, which was added to increase the water solubility, for 16 h to give HE‐M@PDA‐Pc. The loading of Pc was estimated to be 50% by electronic absorption spectroscopy. Finally, this nanocomposite (100 µg mL^−1^ with 2.5 µm Pc) was further treated with the QRH peptide^[^ ^19^ ^]^ (2 µm) in deionized water for 8 h to afford the target multifunctional nanosystem HE‐M@PDA‐Pc‐QRH (Figure S2, Supporting Information).

**Figure 2:**
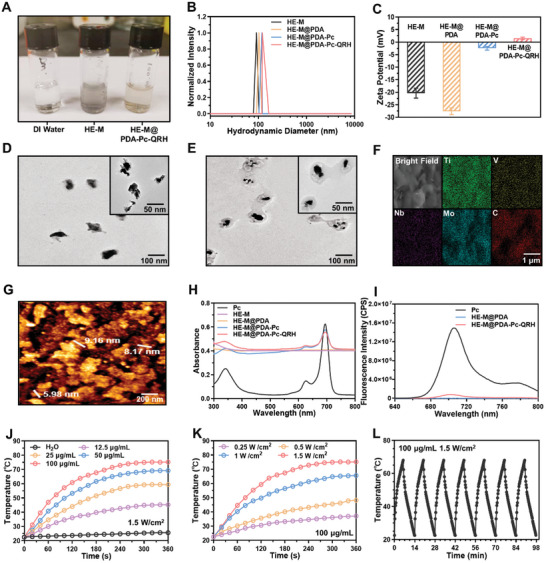
A) A photograph showing the appearance of HE‐M and HE‐M@PDA‐Pc‐QRH dispersed in deionized water. B) Normalized hydrodynamic diameters and C) zeta potentials (*n* = 3) of HE‐M, HE‐M@PDA, HE‐M@PDA‐Pc, and HE‐M@PDA‐Pc‐QRH dispersed in deionized water. TEM images of D) HE‐M and E) HE‐M@PDA‐Pc‐QRH. F) EDS and G) AFM images of HE‐M@PDA‐Pc‐QRH. H) UV–Vis spectra of the various nanocomposites and free Pc in deionized water with 0.5% (v/v) Tween 20 (100 µg mL^−1^ or 2.5 µm of Pc). I) Fluorescence spectra of HE‐M@PDA, HE‐M@PDA‐Pc‐QRH, and free Pc in deionized water with 0.5% (v/v) Tween 20 (100 µg mL^−1^ or 2.5 µm of Pc) (*λ*_ex_ = 610 nm). J) Concentration‐dependent and K) laser power‐dependent photothermal effects of HE‐M@PDA‐Pc‐QRH in deionized water upon irradiation with an 808 nm laser. L) Temperature cycling curve for HE‐M@PDA‐Pc‐QRH in deionized water (100 µg mL^−1^). Each cycle involves laser irradiation (808 nm, 1.5 W cm^−2^) for 6 min followed by natural cooling for 8 min.

These HE‐M nanosheets could be dispersed in water displaying a pale color, and the dispersibility was improved upon coating with PDA (**Figure** **2**A). The intensity‐averaged hydrodynamic diameters of all these nanocomposites were measured by DLS (Figure 2B). The value was generally larger for the PDA‐coated nanosheets. For HE‐M@PDA‐Pc‐QRH, the value was determined to be 118.2 ± 8.3 nm, which was significantly larger than that of the uncoated HE‐M (94.8 ± 3.6 nm). While the zeta potential remained negative for HE‐M@PDA, the surface negative charge was significantly reduced upon conjugation with Pc and QRH (Figure 2C). Figure 2D,E shows the transmission electron microscopy (TEM) images of HE‐M and HE‐M@PDA‐Pc‐QRH, respectively. Comparison of their morphology clearly identified the PDA shell in the latter. The observed dimension of these nanosheets (ca. 100 nm) was in good agreement with that determined by DLS. The elemental composition of HE‐M@PDA‐Pc‐QRH was further confirmed by scanning electron microscopy (SEM) with energy‐dispersive X‐ray spectroscopy (EDS) mapping (Figure 2F). The atomic force microscopy (AFM) images of this nanocomposite were also recorded. A representative image is given in Figure 2G, which showed that the thickness of the nanosheets was 7.8 ± 2.6 nm.

Figure 2H shows the electronic absorption spectra of all these nanocomposites in deionized water in the presence of 0.5% (v/v) Tween 20. The spectrum of free Pc is also included for comparison. As expected, the Q band of Pc at ca. 690 nm could only be observed in the spectra of the two Pc‐containing nanosystems, namely HE‐M@PDA‐Pc and HE‐M@PDA‐Pc‐QRH. The absorption bands were significantly broadened and weaker compared with that of free Pc. Upon excitation at 610 nm, while a strong fluorescence band was observed at 705 nm for free Pc, the emission was largely reduced for HE‐M@PDA‐Pc‐QRH (Figure 2I). Without the Pc component, HE‐M@PDA did not give a noticeable signal. As zinc(II) phthalocyanines are also well‐known photosensitizers for photodynamic therapy,^[^ ^21^ ^]^ the singlet oxygen generation efficiency of HE‐M@PDA‐Pc‐QRH was also determined using 1,3‐diphenylisobenzofuran (DPBF) as the singlet oxygen scavenger. Its decay upon light irradiation (*λ*_ex_ = 610 nm) was monitored spectroscopically at 415 nm along with time in deionized water with 10% (v/v) dimethylsulfoxide (DMSO) added to increase the solubility of DPBF. The results were compared with those in neat water and in the presence of HE‐M or free Pc instead (Figure S3, Supporting Information). The results showed that singlet oxygen could be generated by HE‐M@PDA‐Pc‐QRH. However, while free Pc could consume virtually all DPBF upon irradiation for 30 s, HE‐M@PDA‐Pc‐QRH required 16 min of irradiation to achieve the same effect, showing that the rate of decay of DPBF was much slower than that for free Pc (by ca. 30‐fold), and the singlet oxygen generation efficiency of HE‐M@PDA‐Pc‐QRH was largely reduced. As expected, the change in absorbance of the DPBF's absorption at 415 nm was negligible in neat water and in the presence of non‐Pc‐containing HE‐M. The remarkable reduction in fluorescence emission and singlet oxygen generation could be attributed to the quenching by the HE‐M core^[^ ^22^ ^]^ and the PDA coating,^[^ ^23^ ^]^ as well as the self‐quenching effect of the stacked Pc molecules on the nanosheets.^[^ ^24^ ^]^

The photothermal property of HE‐M@PDA‐Pc‐QRH was then studied under different conditions. Figure 2J shows its photothermal response at different concentrations (up to 100 µg mL^−1^) upon 808 nm laser irradiation at a power of 1.5 W cm^−2^. It can be seen that the rise in temperature was generally enhanced with the concentration of the nanosheets. Similarly, the effect of laser power was also studied. At a fixed concentration of 100 µg mL^−1^, the effect was also significantly increased with the power of the laser (0.25‐1.5 W cm^−2^) (Figure 2K). Over a period of 6 min, the temperature could increase up to 76 °C at the maximum concentration (100 µg mL^−1^) and laser power (1.5 W cm^−2^) used. Under these conditions and upon irradiation for 6 min followed by natural cooling for 8 min, a cycle of the temperature change was observed. As shown in Figure 2L, the cycle could be repeated perfectly for at least 7 times, showing that the material was highly thermally stable.

For comparison, the temperature rise profiles of the other HE‐M‐based nanosystems, including HE‐M, HE‐M@PDA, and HE‐M@PDA‐Pc were also recorded under the same conditions (Figure S4, Supporting Information). The virtually identical response showed that the photothermal effect was mainly caused by the HE‐M core, and the contribution of the other components, including the PDA layer, was negligible. Moreover, we also compared the photothermal effect of HE‐M with that of some well‐known 2D materials, including Ti_3_C_2_, Nb_2_C, and black phosphorus. As shown in Figure S5 (Supporting Information), HE‐M exhibited the best performance. The photothermal conversion efficiency (η) was determined by the method reported by Wang et al.^[^ ^25^ ^]^ Figure S6A (Supporting Information) shows the natural cooling curve for HE‐M after reaching the maximum temperature upon laser irradiation at 808 nm (1.5 W cm^−2^), from which the heat dissipation time constant (τ_s_) was determined to be 133 s based on the linear plot of time (*t*) against – ln ($\frac{T-T\mathrm{surr}}{T\max-T\mathrm{surr}}$) (Figure S6B, Supporting Information), where *T* is the temperature at time *t*, *T*surr is the ambient temperature of the surrounding, and *T*max is the equilibrium temperature. By substituting all the relevant parameters, the value of η was determined to be 56.1%, which was significantly higher than that of the other materials [49.6% (for Ti_3_C_2_), 38.3% (for Nb_2_C), and 37.6% (for black phosphorus)] determined by the same method. All these results showed that the HE‐M nanosheets would be an excellent photosensitizer for PTT.

### 2.2 Cellular Uptake, Biocompatibility, and Photocytotoxicity

**Figure 3:**
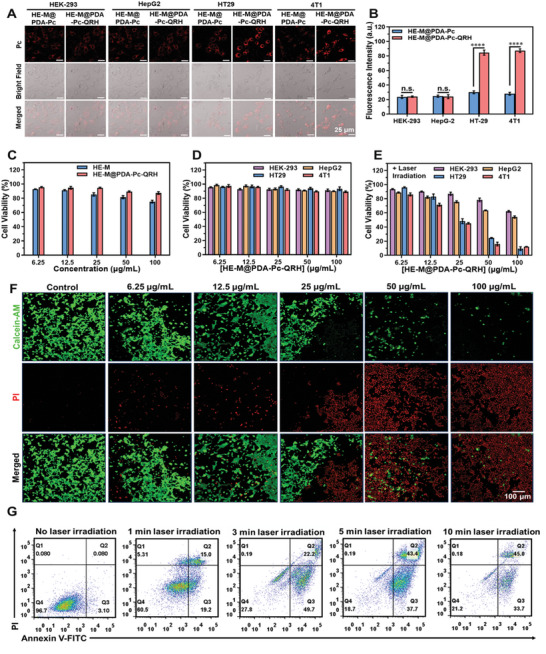
A) Confocal images of HEK‐293, HepG2, HT29, and 4T1 cells after incubation with HE‐M@PDA‐Pc or HE‐M@PDA‐Pc‐QRH (10 µg mL^−1^) for 8 h. Scale bar = 25 µm. B) Corresponding quantified intracellular fluorescence intensities (*n* = 50 cells). Data are reported as the mean ± standard deviation (SD) for three independent experiments. n.s., not significant; ****p < 0.0001. C) Cell viabilities of 4T1 cells after incubation with various concentrations of HE‐M or HE‐M@PDA‐Pc‐QRH for 8 h. Cell viabilities of HEK‐293, HepG2, HT29, and 4T1 cells after incubation with various concentrations of HE‐M@PDA‐Pc‐QRH for 8 h in the D) absence and E) presence of laser irradiation at 808 nm (1 W cm^−2^) for 10 min. Data are reported as the mean ± SD for four independent experiments. F) Fluorescence imaging of 4T1 cells after the above treatment and staining with calcein‐AM (green, live cells) and PI (red, dead cells). Scale bar = 100 µm. G) Flow cytometric analysis of 4T1 cells after incubation with HE‐M@PDA‐Pc‐QRH (100 µg mL^−1^) for 8 h, followed by laser irradiation at 808 nm (1 W cm^−2^) for 1, 3, 5, and 10 min, using a Dead Cell Apoptosis kit with Annexin V‐FITC and PI.

The cellular uptake of HE‐M@PDA‐Pc‐QRH was then examined by monitoring the intracellular fluorescence of Pc released inside the cells. 4T1 Murine mammary cancer cells, which are known to have high expression of EGFR,^[^ ^26^ ^]^ were first used for this study. Upon incubation with different concentrations of these nanosheets (10, 20, and 40 µg mL^−1^) for different periods of time (1, 4, 8, and 12 h), the confocal images of the cells were captured. The results showed that while the intracellular fluorescence intensity generally increased with the concentration and the incubation time, the intensity was already notable after incubation with 10 µg mL^−1^ of the nanosheets for 8 h (Figure S7, Supporting Information). Hence, these conditions were used for further study of the cell selectivity. For this study, apart from 4T1 cells, another EGFR‐positive HT29 human colorectal adenocarcinoma cells^[^ ^27^ ^]^ and EGFR‐negative HEK‐293 human embryonic kidney normal cells and HepG2 human hepatocarcinoma cells^[^ ^28^ ^]^ were also selected, using the non‐QRH‐conjugated nanosystem HE‐M@PDA‐Pc for comparison. The cells were incubated with these nanosheets (10 µg mL^−1^) for 8 h, and then their confocal images were recorded. As shown in **Figure** **3**A, the fluorescence remained weak for HE‐M@PDA‐Pc in all the four cell lines. In contrast, the QRH‐conjugated counterpart showed a cell‐selective property. While the intracellular fluorescence intensity was comparable with that for HE‐M@PDA‐Pc against HEK‐293 and HepG2 cells, the intensity was significantly higher for HT29 and 4T1 cells. The increment was about 3‐fold based on the quantified intracellular fluorescence intensity (Figure 3B). The enhanced cellular uptake by these cells could be attributed to the EGFR‐mediated endocytosis. The results also provided evidence that the QRH peptide had been successfully conjugated to the nanosheets.

The biocompatibility of HE‐M and HE‐M@PDA‐Pc‐QRH was then briefly evaluated using 4T1 cells. After incubation with various concentrations (up to 100 µg mL^−1^) of these nanosheets for 8 h, the cell viability was determined by MTT assay [MTT = 3‐(4,5‐dimethylthiazol‐2‐yl)‐2,5‐diphenyltetrazolium bromide] (Figure 3C). For HE‐M, the cell viability decreased slightly along with the concentration of the nanosheets, and the value reached ca. 70% at a drug dose of 100 µg mL^−1^. With a coating of PDA, HE‐M@PDA‐Pc‐QRH exhibited significantly lower cytotoxicity, showing that this nanocomposite had improved biocompatibility. This property was further evaluated against the other three cell lines mentioned above. As shown in Figure 3D, HE‐M@PDA‐Pc‐QRH was generally highly compatible to all these cell lines. In contrast, upon laser irradiation (808 nm, 1 W cm^−2^, 10 min), HE‐M@PDA‐Pc‐QRH exhibited concentration‐dependent cytotoxicity (Figure 3E), which could be attributed to the photothermal effect of the HE‐M core. As expected, the cytotoxicity was significantly higher for the two EGFR‐positive cell lines because of the enhanced uptake. At a drug dose of 100 µg mL^−1^, the nanocomposite could eliminate ca. 90% of the HT29 and 4T1 cells, while over 60% of the HEK‐293 and HepG2 cells remained alive after the treatment. The half‐maximal inhibitory concentrations (IC_50_ values) for HT29 and 4T1 cells were determined to be about 25 µg mL^−1^. Since the singlet oxygen generation ability of Pc was largely quenched after immobilization on the surface of HE‐M@PDA as mentioned above and the focus of this study was on the therapeutic effect of HE‐M arising from its superior photothermal property, the photodynamic effect of this nanosystem induced by exciting Pc at ca. 690 nm was not investigated.

In addition, the elimination of 4T1 cells by the photothermal effect of HE‐M@PDA‐Pc‐QRH was further examined using a live/dead double staining protocol based on calcein acetoxymethyl ester (calcein AM) and propidium iodide (PI). After incubation with various concentrations of these nanosheets (up to 100 µg mL^−1^) for 8 h, followed by the light treatment (808 nm, 1 W cm^−2^, 10 min), the cells were stained with these dyes, and then their confocal fluorescence images were recorded. As shown in Figure 3F, the number of dead cells as indicated by red fluorescence increased significantly when a higher concentration of the nanosheets was used, reflecting a higher extent of cell killing.

Finally, the cell death mechanism induced by this nanocomposite was examined using a Dead Cell Apoptosis kit with Annexin V‐FITC and PI. After laser irradiation at 808 nm (1 W cm^−2^) for 1 min, early apoptotic (quadrant Q3) and late apoptotic (quadrant Q2) cells already appeared. The percentage increased significantly from 19.2% to 49.7% for early apoptotic cells and from 15.0% to 22.2% for late apoptotic cells upon prolonging the irradiation time to 3 min. Extending the irradiation time to 5 min led to more late apoptotic cells (43.4%), and the total percentage of early and late apoptotic cells increased further from 71.9% to 81.1%. Further extending the irradiation time to 10 min did not significantly change the flow cytometric results. The results showed that the photothermal killing of cells was mainly through apoptosis, which is a common cell‐death pathway of MXene‐based PTT.^[^ ^29^ ^]^

### 2.3 In Vivo Fluorescence and Photothermal Imaging

**Figure 4:**
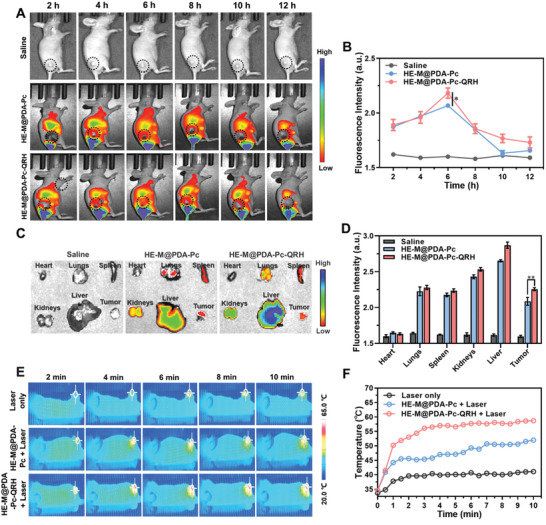
A) In vivo fluorescence images of 4T1 tumor‐bearing mice after intravenous injection with saline (200 µL) with or without the presence of HE‐M@PDA‐Pc or HE‐M@PDA‐Pc‐QRH (200 µg) recorded after different periods of time. The apparently extremely high fluorescence intensity at the bottom part is just an artifact. B) Change in the corresponding quantified fluorescence intensity per unit area of the tumor with time. Data are reported as the mean ± SD (*n* = 4). *p < 0.05. C) Representative fluorescence images of some major organs and the tumor harvested at 6 h post‐injection. D) Corresponding quantified fluorescence intensities per unit area. Data are reported as the mean ± SD (*n* = 3). **p < 0.01. E) Representative in vivo infrared thermal images of one of the mice from each of the above three groups recorded upon laser irradiation at 808 nm at the tumor at 6 h post‐injection for different periods of time. F) Change in the temperature at the tumor with irradiation time for the three mice.

To examine the tumor‐directing effect of the QRH peptide at the animal level, we established a 4T1 tumor‐bearing mouse model and compared the in vivo fluorescence and photothermal imaging effects of HE‐M@PDA‐Pc and HE‐M@PDA‐Pc‐QRH. In this experiment, the mice were intravenously injected with saline (200 µL) with or without the presence of one of these nanosystems (200 µg). The images of the mice based on the fluorescence of Pc were then captured at different time points over a period of 12 h using an in vivo imaging system. **Figure** **4**A shows the representative images for each group (*n* = 4). While fluorescence could hardly be seen for the saline‐treated mice, strong fluorescence was observed in the body of the mice being treated with the two nanosystems. The average fluorescence intensity per unit area of the tumor was also determined for each data point. Figure 4B depicts the results for all the three groups over this period of time. It can be seen that the values were generally much higher for the two nanosystems compared with the saline control group. The tumor‐localization property of these nanosheets could be attributed to the enhanced permeability of retention effect arising from their nanoscale property.^[^ ^30^ ^]^ For the two nanosystems, the quantified intensity reached the maximum after 6 h. The values for HE‐M@PDA‐Pc‐QRH were slightly higher than those for the non‐QRH‐conjugated counterpart, particularly at 6 and 10 h post‐injection, which could be ascribed to the tumor‐directing effect of QRH.

To further reveal the biodistribution of these nanosystems, an ex vivo study was also performed by analyzing the fluorescence intensities of the tumor and major organs, including heart, lungs, spleen, kidneys, and liver harvested at 6 h post‐injection. Figure 4C,D shows the corresponding representative fluorescence images and quantified fluorescence intensities per unit area, respectively. The results showed that while HE‐M@PDA‐Pc and HE‐M@PDA‐Pc‐QRH spread to the lungs, spleen, kidneys, and liver, the localization in tumor was also substantial, and the extent was significantly higher for the QRH‐conjugated nanosystem.

Apart from using the fluorescence of Pc, the photothermal effect of the HE‐M core of these nanosystems could also be used for in vivo imaging. Figure 4E shows the representative infrared thermal images of one of the mice from each of the above three groups recorded upon laser irradiation at 808 nm at the tumor at 6 h post‐injection for different periods of time, while Figure 4F depicts the corresponding variation of the temperature at the tumor site with the irradiation time. It can be seen that the increase in temperature upon laser irradiation followed the trend: HE‐M@PDA‐Pc‐QRH > HE‐M@PDA‐Pc > saline. The higher temperature at and brighter image of the tumor for HE‐M@PDA‐Pc‐QRH compared with those for the non‐QRH‐conjugated nanosystem could be attributed to the QRH‐promoted tumor localization. The results of both imaging modalities showed that the immobilized QRH peptide could promote the accumulation of the HE‐M@PDA nanosheets in the tumor.

### 2.4 In Vivo Antitumor Effect

**Figure 5:**
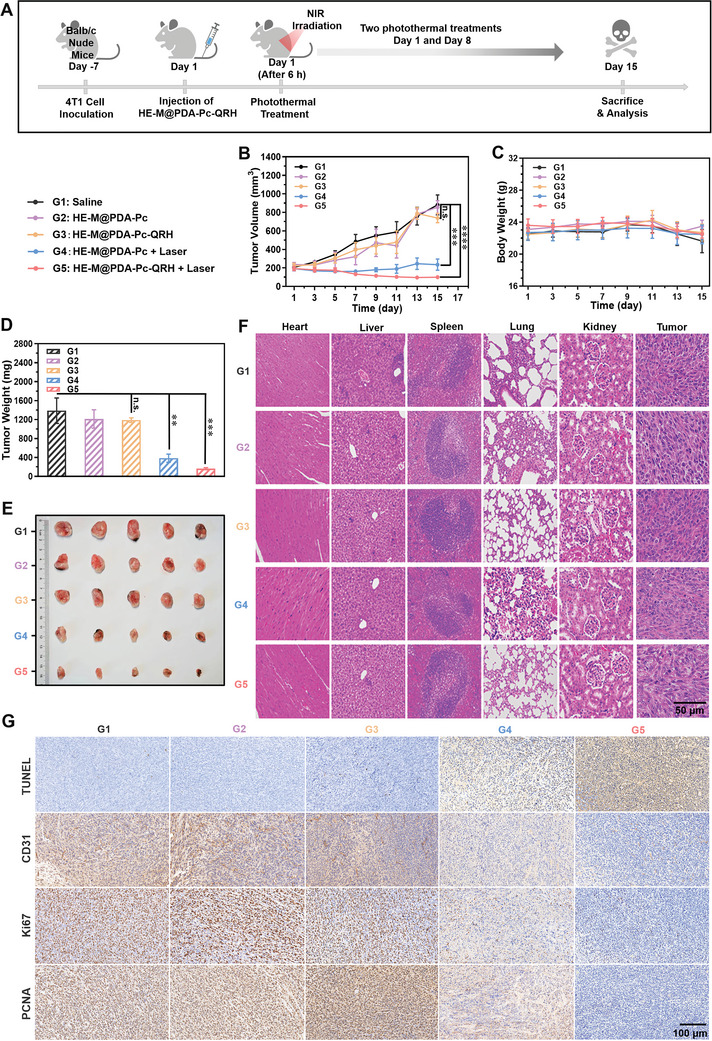
A) Timeline of the in vivo study of the PTT efficacy of HE‐M@PDA‐Pc‐QRH against 4T1 tumor‐bearing nude mice. B) Tumor growth curves for the mice after receiving different treatments: G1: intravenous injection with saline (200 µL); G2: intravenous injection with HE‐M@PDA‐Pc in saline (200 µg, 200 µL); G3: intravenous injection with HE‐M@PDA‐Pc‐QRH in saline (200 µg, 200 µL); G4: intravenous injection with HE‐M@PDA‐Pc in saline (200 µg, 200 µL), followed by laser treatment (808 nm, 1 W cm^−2^, 10 min) at 6 h post‐injection and on day 8; G5: intravenous injection with HE‐M@PDA‐Pc‐QRH in saline (200 µg, 200 µL), followed by laser treatment (808 nm, 1 W cm^−2^, 10 min) at 6 h post‐injection and on day 8. C) Changes in the body weights of the mice for G1 to G5; D) Weights and E) sizes of the tumors harvested from the different groups of the mice on day 15 (*n* = 5). B–D) Data are reported as the mean ± SD (*n* = 5). n.s., not significant; **p < 0.01; ***p < 0.001; and ****p < 0.0001. F) H&E‐stained images of different organ and tumor slides from the mice scarified on day 15 after different treatments. G) Representative immunohistochemical images of the tumor sections prepared at the end of the above treatments and stained for TUNEL assay and detection of CD31, Ki67, and PCNA. Scale bar = 100 µm.

The in vivo PTT efficacy of HE‐M@PDA‐Pc‐QRH was then evaluated using 4T1 tumor‐bearing nude mice. The protocol is shown in **Figure** **5**A. In short, 4T1 cells were first injected subcutaneously into a group of nude mice. After 7 d when the tumors reached a volume of ca. 150 mm^3^, a dispersion of HE‐M@PDA‐Pc‐QRH in saline (200 µg, 200 µL) was injected intravenously. After 6 h, the tumor region was irradiated with an 808 nm laser (1 W cm^−2^) for 10 min. This photothermal treatment was repeated on day 8 to enhance the treatment effect. For comparison, various control groups were also used, which included G1: intravenous injection with saline; G2: intravenous injection with HE‐M@PDA‐Pc in saline; G3: intravenous injection with HE‐M@PDA‐Pc‐QRH in saline; and G4: intravenous injection with HE‐M@PDA‐Pc in saline followed by repeated laser treatment. The tumor volumes were then monitored for each group of the mice over a period of 15 d. As shown in Figure 5B, while the size continued to grow for control groups G1‐G3, the treatments for G4 and G5 were very effective in the inhibition of the tumor growth. For G5, the tumors could virtually be eliminated. During the course of all these treatments, the change in the body weight of the mice was negligible except for G1, for which the average weight was slightly decreased after day 11 (Figure 5C), which could be ascribed to the continuous growth of tumor that affected the health of the mice. At the end of the experiment, the tumors were harvested. Both the weight (Figure 5D) and size (Figure 5E) variations were consistent with the tumor‐inhibition curves in Figure 5B. Apart from the tumors, the major organs, including heart, liver, spleen, lung, and kidney were also harvested from the mice. Hematoxylin and eosin (H&E) staining showed that all the five treatments did not induce noticeable tissue damage to these organs. For the tumor sections from the treatment groups G4 and G5, they displayed more necrotic and apoptotic cells compared with those from the other three control groups (Figure 5F). In addition, the tumors were subjected to further immunohistochemical evaluation of the extent of apoptotic DNA fragmentation by TUNEL assay, the angiogenesis marker CD31, and the cell proliferation markers Ki67 and proliferating cell nuclear antigen (PCNA). As shown in Figure 5G, compared with the tumor sections for other groups, those for G5 showed more extensive cell apoptosis and reduced angiogenesis and cell proliferation. All these results clearly showed that both HE‐M@PDA‐Pc and HE‐M@PDA‐Pc‐QRH exhibited a strong photothermal effect upon laser irradiation that can effectively eliminate the tumor. Having immobilized QRH, which could promote the tumor localization, the latter was particularly potent. The high biocompatibility of these PDA‐coated nanosystems also resulted in minimal side effects.

### 2.5 Systemic Toxicity

**Figure 6:**
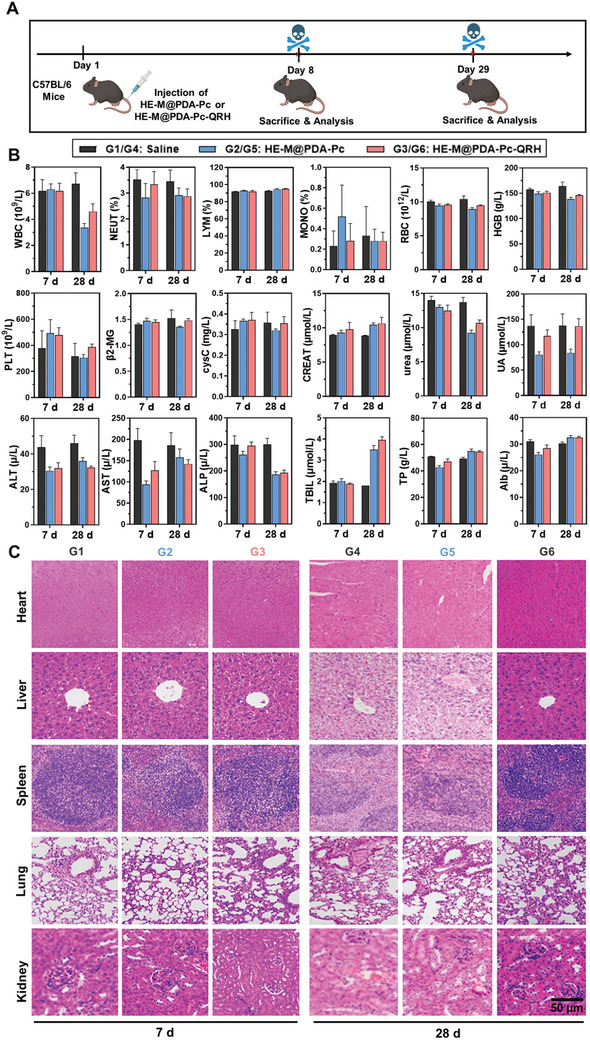
A) Timeline of the in vivo study of the systemic toxicity of HE‐M@PDA‐Pc and HE‐M@PDA‐Pc‐QRH against C57BL/6 mice. B) Measurements of the hematologic parameters and blood and urine biochemical indexes for the renal and liver functions of the mice being intravenously injected with saline (200 µL) with or without the presence of HE‐M@PDA‐Pc or HE‐M@PDA‐Pc‐QRH (200 µg), followed by housing for 7 or 28 d. Data are reported as the mean ± SD (*n* = 10). C) H&E‐stained images of different organ slides from the mice scarified on day 8 or 29 after the above treatments.

Finally, both the short‐term and long‐term systemic toxicity of HE‐M@PDA‐Pc and HE‐M@PDA‐Pc‐QRH was examined in detail according to the timeline shown in **Figure** **6**A to reveal their potential for biomedical applications. For this study, C57BL/6 mice were selected and randomly divided into six groups (*n* = 10), namely G1/G4: a control group being housed for 7/28 d; G2/G5: the mice were intravenously injected with HE‐M@PDA‐Pc in saline (200 µg, 200 µL) before being housed for 7/28 d; and G3/G6: the mice were intravenously injected with HE‐M@PDA‐Pc‐QRD in saline (200 µg, 200 µL) before being housed for 7/28 d. The routine hematologic parameters of these mice were then measured, including the white blood cells (WBC), neutrophils (NEUT), lymphocytes (LYM), monocytes (MONO), red blood cells (RBC), hemoglobin (HGB), and platelets (PLT) (Figure 6B). It was found that the levels of these parameters were generally not significantly changed upon treatment with these nanosystems after 7 and 28 d (except for WBC after 28 d). These results showed that they did not cause noticeable hematological toxicity to the mice. In addition, we also measured some standard blood and urine biochemical indexes for the renal functions, including β2‐microglobulin (b2‐MG), cystatin C (cysC), creatinine (CREAT), urea, and uric acid (UA), as well as for the liver functions, including alanine transaminase (ALT), aspartate transaminase (AST), alkaline phosphatase (ALP), total bilirubin (TBIL), total protein (TP), and albumin (Alb). As shown in Figure 6B, the levels of ALT, AST, and ALP were slightly lower after the treatment of the nanosystems, while the TBIL level increased significantly after 28 d. The treatment with HE‐M@PDA‐Pc‐QRH also led to significant reduction in UA. Other biochemical indexes did not change significantly. The overall results indicated that both nanosystems did not induce noticeable liver and nephro‐toxicity to the mice. To further confirm this point, the heart, liver, spleen, lung, and kidney of the mice were sectioned and subjected to H&E staining. As shown in Figure 6C, no noticeable damage was detected in all the tissues, even after 28 d, which further demonstrated the high biocompatibility of these HE‐M nanosheets.

## 3 Conclusion

HE‐Ms are highly promising 2D materials possessing a range of intriguing properties. Their applications in the biomedical arena, however, are restricted to photo‐ and sono‐elimination of bacteria so far. We report herein an extended application of this class of materials in targeted anticancer PTT, utilizing the superior photothermal property of these materials and the high biocompatibility and versatility of PDA. HE‐M nanosheets of the formula TiVNbMoC_3_T*_x_* were first coated with a layer of PDA formed by self‐polymerization of dopamine. It was then followed by further immobilization of a Pc‐based fluorophore for bioimaging and the QRH peptide for targeting the EGFR overexpressed in cancer cells. The resulting PDA‐coated nanosheets exhibited an extraordinary photothermal effect with a light‐to‐heat conversion efficiency of 56.1%, which is significantly higher than that of conventional 2D materials. In vitro studies showed that they could be selectively internalized into EGFR‐positive 4T1 and HT29 cancer cells. While they were highly biocompatible in the absence of light irradiation, they became highly cytotoxic upon laser irradiation at 808 nm with an IC_50_ value of about 25 µg mL^−1^, killing the cells mainly through apoptosis. The presence of a HE‐M core and Pc also enabled the nanosystems to be used for in vivo infrared thermal and fluorescence imaging. Using a 4T1 tumor‐bearing mouse model, we also demonstrated that these tailor‐fabricated nanosystems could effectively eliminate the tumor through PTT without causing noticeable side effect and systemic toxicity to the mice. The overall results showed that these HE‐M nanosheets exhibited a great potential for anticancer application, and the versatile PDA coating could facilitate the incorporation of various functional units to further enhance their theranostic efficacy.

## 4 Experimental Section

### General

All reagents and solvents were of reagent grade and used as received. Pc^[^ ^20^ ^]^ and QRH peptide^[^ ^19^ ^]^ were prepared according to the previously described procedure. DLS was performed using a DelsaMax Pro analyzer. TEM images were recorded on a FEI Tecnai G2 Spirit transmission electron microscope operated at 120 kV acceleration voltage. The morphology and elemental composition were examined using a JEOL JSM7800‐F scanning electron microscope. A Digital Instruments multimode scanning probe microscope was used to detect the thickness of the nanocomposites. UV‐Vis and steady‐state fluorescence spectra were recorded with a Cary 5G UV‐Vis‐NIR spectrophotometer and a Hitachi F‐7000 spectrofluorometer, respectively.

### Preparation of HE‐M Nanosheets with a Suitable Size

HE‐M of the formula TiVNbMoC_3_T_x_ was purchased from Beike Nano Technology (Suzhou, China). A dispersion of this material with an initial diameter of ca. 1 µm in deionized water in a concentration of 1 mg mL^−1^ was sonicated (0.46 kW h^−1^) at room temperature for 24 h. The mixture was then centrifuged at 9000 rpm for 20 min. The supernatant was collected and then centrifuged again at 12 000 rpm for a further 20 min. The resulting precipitate was collected as Sample 1. The supernatant was then centrifuged again at 14 500 rpm for 20 min. The precipitate collected was regarded as Sample 2, while the supernatant was named as Sample 3. The hydrodynamic diameters of these batches of nanosheets were determined by DLS (Figure S1, Supporting Information), and Sample 2 was selected for further modification.

### Preparation of HE‐M@PDA, HE‐M@PDA‐Pc, and HE‐M@PDA‐Pc‐QRH

A dispersion of HE‐M nanosheets (Sample 2) in deionized water (100 µg mL^−1^, 1 mL) was centrifuged at 12 000 rpm for 20 min. The precipitate collected was then mixed with a solution of dopamine in deionized water (1 mg mL^−1^, 0.1 mL) and Tris‐HCI buffer pH 8.5 (10 mM) (0.9 mL). After sonication in a water bath at room temperature for 3 h, the mixture was centrifuged at 12 000 rpm for 20 min. The resulting precipitate was collected, which was then washed with deionized water and centrifuged thrice to give HE‐M@PDA.

Deionized water (1 mL) was then added to this precipitate, and the dispersion was centrifuged at 12 000 rpm for 10 min. The precipitate collected was then mixed with a solution of Pc in deionized water in the presence of 0.5% (v/v) Tween 20 (5 µm, 1 mL). The mixture was rotatory mixed at room temperature for 16 h. After centrifugation at 12 000 rpm for 5 min, the precipitate was collected as HE‐M@PDA‐Pc. Based on the Q‐band absorbance of the supernatant, the amount of free Pc could be determined, from which the Pc loading was estimated to be 50%, i.e., 2.5 µm Pc immobilized on the nanosheets of HE‐M@PDA (at 100 µg mL^−1^). The isolated HE‐M@PDA‐Pc was then mixed with a solution of QRH in deionized water (2 µm, 1 mL) by a rotator at room temperature for 8 h to give HE‐M@PDA‐Pc‐QRH, which was collected by centrifugation. The procedure for preparing these nanosystems is also depicted in Figure S2, Supporting Information.

### Photothermal Property of HE‐M@PDA‐Pc‐QRH

The photothermal response of this nanocomposite in deionized water was triggered by irradiation with an 808 nm laser. The increase in temperature at different concentrations of the nanosheets and under different powers of laser irradiation was monitored over a period of 6 min. The light‐to‐heat conversion efficiency was determined according to the method described previously.^[^ ^25^ ^]^ Comparison was made with the classical 2D materials, including Ti_3_C_2_, Nb_2_C, and black phosphorus.

### Singlet Oxygen Generation of HE‐M@PDA‐Pc‐QRH

A dispersion of these nanosheets in deionized water (80 µg mL ^−1^ or 2 µm of Pc) in the presence of DPBF (90 µm) and 10% (v/v) DMSO was irradiated with red light from a 200 W halogen lamp after passing through a water tank for cooling and a color glass filter (Newport) cut‐on at 610 nm. The absorbance at 415 nm was monitored during the course of irradiation for 16 min. For comparison, mixtures containing HE‐M, free Pc, or neat water were also studied. For the mixture containing free Pc, due to its high singlet oxygen generation efficiency, the mixture was irradiated only for 30 s.

### Cell Lines and Culture Conditions

4T1 (ATCC, no. CRL‐2539) and HT29 (ATCC, no. HTB‐38) cells were maintained in Roswell Park Memorial Institute (RPMI) 1640 medium (Invitrogen, no. 23400‐021) supplemented with fetal bovine serum (FBS) (10%) (ThermoFisher Scientific, no. 10270‐106) and a penicillin–streptomycin solution (100 unit mL^−1^ and 100 µg mL^−1^, respectively). HEK‐293 (ATCC, no. CRL‐1573) and HepG2 (ATCC, no. HB‐8065) cells were maintained in Dulbecco's modified Eagle medium (DMEM) (ThermoFisher Scientific, no. 12100‐046) supplemented with FBS (10%) and a penicillin–streptomycin solution (100 unit mL^−1^ and 100 µg mL^−1^, respectively). All the cells were grown at 37 °C in a humidified 5% CO_2_ atmosphere.

### Cellular Uptake

Approximately 2 × 10^5^ cells in the corresponding medium were seeded on a confocal dish and incubated overnight at 37 °C under 5% CO_2_. To study the optimized incubation conditions, 4T1 cells were incubated with different concentrations (10, 20, and 40 µg mL^−1^) of HE‐M@PDA‐Pc‐QRH in RPMI 1640 medium for 1, 4, 8, and 12 h. The cells were then rinsed with phosphate‐buffered saline (PBS) twice before being examined with a Leica TCS SP8 high‐speed confocal microscope equipped with a 638 nm laser. The fluorescence due to Pc was monitored at 650−750 nm for all these conditions. The images were digitized and analyzed using Leica Application Suite X software. To study the cell selective property, 4T1, HT29, HEK‐293, and HepG2 cells were incubated with HE‐M@PDA‐Pc or HE‐M@PDA‐Pc‐QRH in the corresponding medium (10 µg mL^−1^) for 8 h. The fluorescence images were then recorded, and the quantified fluorescence intensities were determined using the same confocal microscope.

### In Vitro Dark and Photo‐Cytotoxicity

Approximately 1 × 10^4^ cells per well in the corresponding medium were inoculated in 96‐well plates and incubated overnight at 37 °C in a humidified 5% CO_2_ atmosphere. The culture medium was then replaced with a fresh medium containing HE‐M or HE‐M@PDA‐Pc‐QRH at various concentrations (6.25 to 100 µg mL^−1^). After incubation for 8 h, the cells were rinsed with 100 µL of PBS twice and replenished with 100 µL of fresh medium. They were then incubated at 37 °C for 2 h with or without prior laser irradiation at 808 nm (1 W cm^−1^) for 10 min. The cell viabilities were then measured using a MTT assay. In this assay, a solution of MTT (Sigma) in PBS (3 mg mL^−1^, 50 µL) was added to each well. After incubation for 4 h, 100 µL of DMSO was added to each well and the plates were placed on a Bio‐Rad microplate reader to detect the absorbance at 490 nm. The average absorbance of the blank wells (not seeded with cells) was subtracted from the readings of the other wells. The cell viability was determined by the equation: %Viability = [∑(*A*_i_/*A*_control_ × 100)]/*n*, where *A*_i_ is the absorbance of the *i* ^th^ datum (*i* = 1, 2, …, *n*), *A*_control_ is the average absorbance of the control wells in which the nanosystem was absent, and *n* (= 4) is the number of data points.

The photocytotoxicity of HE‐M@PDA‐Pc‐QRH was further assessed using a live/dead double staining protocol based on calcein AM and PI. In this study, 4T1 cells were incubated with different concentrations of HE‐M@PDA‐Pc‐QRH (6.25 to 100 µg mL^−1^) for 8 h, followed by laser irradiation at 808 nm (1 W cm^−1^) for 10 min. After incubation at 37 °C for 2 h, the cells were rinsed with PBS for three times, followed by the treatment with calcein‐AM and PI (Dojindo Laboratories, Kumamoto, Japan) for 30 min according to the manufacturer's instructions. The live cells (in green fluorescence) and dead cells (in red fluorescence) were detected using an Olympus IX71 inverted fluorescence microscope. Calcein‐AM was excited at 494 nm, and its fluorescence was monitored at 510–540 nm. For PI, it was excited at 535 nm, and its fluorescence was monitored at 610–635 nm.

### Cell Death Mechanism

Approximately 2 × 10^5^ 4T1 cells per well in RPMI 1640 medium were seeded in a six‐well plate and incubated overnight at 37 °C in a humidified 5% CO_2_ atmosphere. The culture medium was then replaced with a fresh medium containing HE‐M@PDA‐Pc‐QRH (100 µg mL^−1^), in which the cells were incubated for 8 h. The cells were then rinsed with 100 µL of PBS twice and replenished with 100 µL of fresh medium with or without further laser irradiation at 808 nm (1 W cm^−1^) for 1, 3, 5, and 10 min. After the addition of 0.5 mL of the medium to quench the activity of trypsin, the cell mixture was centrifuged at 1500 rpm for 5 min at room temperature. The cell pellet was washed with 1 mL of PBS and subjected to centrifugation for three times. After resuspending the cells in 1 mL of PBS, the cell death mechanism was determined using a Dead Cell Apoptosis kit with Annexin V‐FITC and PI (Beyotime Biotech., China) according to the manufacturer's instructions. Their intracellular fluorescence intensities were measured using a BD FACSVerse flow cytometer (Becton Dickinson) with 10^4^ cells counted in each sample.

### In Vivo Imaging

All experiments involving live animals were performed in strict accordance with the animal experimentation guidelines and were approved by the Animal Experimentation Ethics Committee of Shenzhen Lingfu Top Biotechnology Co. Ltd. (No. TOP‐IACUC‐2024‐0158). License to conduct animal experiments was obtained from the SPF Experimental Animal Center of Shenzhen Lingfu Top Biotechnology Co. Ltd. All mice were kept under pathogen‐free conditions with free access to food and water.

For the in vivo fluorescence imaging study, subcutaneous breast cancer xenografts were established in nude mice with 4T1 cells. When the tumor volume reached ca. 150 mm^3^, the mice were randomly divided into 3 groups (*n* = 4) receiving the following treatments. G1: intravenous injection with saline (200 µL); G2: intravenous injection with HE‐M@PDA‐Pc (200 µg, 200 µL); and G3: intravenous injection with HE‐M@PDA‐Pc‐QRH (200 µg, 200 µL). After that, the fluorescence images of the mice were monitored over a period of 12 h using a Xenogen IVIS Spectrum imaging system (excitation wavelength = 610 nm and emission wavelength = 610–700 nm). For the ex vivo study, the tumors and major organs, including heart, lungs, spleen, kidneys, and liver were harvested at 6 h post‐injection. Their fluorescence images and intensities were recorded using the same imaging system.

For the in vivo photothermal detection, three groups of mice (*n* = 3) were treated as described above respectively. After 6 h, they were irradiated with an 808 nm laser (1 W cm^−2^) over a period of 10 min. The temperature change at the tumor sites was detected and recorded during the course using an infrared imager (HM‐TPH10‐3AUF, Hikmicrotech).

### In Vivo PTT Efficacy

To establish a subcutaneous breast cancer model, 25 healthy female nude mice (5–6 weeks of age, 16–20 g) were used. Approximately 5 × 10^5^ 4T1 cells were injected subcutaneously into the left side of the mice. When the tumor volume reached ca. 150 mm^3^, the mice were randomly divided into 5 groups (*n* = 5) receiving the following treatments. G1: intravenous injection with saline (200 µL); G2: intravenous injection with HE‐M@PDA‐Pc in saline (200 µg, 200 µL); G3: intravenous injection with HE‐M@PDA‐Pc‐QRH in saline (200 µg, 200 µL); G4: intravenous injection with HE‐M@PDA‐Pc in saline (200 µg, 200 µL) followed by laser irradiation at 808 nm (1 W cm^−1^) for 10 min at 6 h post‐injection and on day 8; and G5: intravenous injection with HE‐M@PDA‐Pc‐QRH in saline (200 µg, 200 µL) followed by laser irradiation at 808 nm (1 W cm^−1^) for 10 min at 6 h post‐injection and on day 8. For G4 and G5, the mice were anesthetized with isoflurane before the laser treatment. The tumor volume as determined by the equation: Tumor volume = (tumor length) × (tumor width)^2^/2 was then measured with a digital caliper every 2 d, and the mice were photographed over a period of 14 d. The mice were then scarified and dissected on day 15. The tumor and major organs, including heart, liver, spleen, lung, and kidney were harvested from the mice and fixed with 4% paraformaldehyde and stained with H&E for histological analysis.

For immunohistochemical analysis, the tumors collected after the above treatments were fixed with 4% paraformaldehyde. To evaluate apoptosis, TUNEL staining was performed according to the manufacturer's instructions of the detection kit (Servicebio, no. G1507). For analysis of the angiogenesis marker CD31 and the cell proliferation markers Ki67 and PCNA, the tumor sections were stained with an anti‐CD31 antibody (Servicebio, no. GB113151, 1:300), anti‐Ki67 antibody (Servicebio, no. GB111499, 1:1000), and anti‐PCNA antibody (Servicebio, no. GB12010, 1:3000), respectively, overnight at 4 °C, followed by incubation with 1× secondary antibody solution (50 µL) for 1 h (Servicebio, no. GB23001, 1:200). The sections were then stained with 3,3′‐diaminobenzidine and hematoxylin for signal detection.

### Systemic Toxicity

Sixty healthy female C57BL/6 mice (5‐6 weeks of age, 16–20 g) were randomly divided into 6 groups (*n* = 10), namely G1/G4: a control group being housed for 7/28 d; G2/G5: the mice were intravenously injected with HE‐M@PDA‐Pc in saline (200 µg, 200 µL) before being housed for 7/28 d; G3/G6: the mice were intravenously injected with HE‐M@PDA‐Pc‐QRD in saline (200 µg, 200 µL) before being housed for 7/28 d. Blood, urine, and major organs, including heart, liver, spleen, lung, and kidney were collected from the mice in different groups on day 7 and 28 after the injection. Liver and kidney function tests, routine blood tests, and H&E staining were performed to assess the systemic toxicity of the two nanosystems.

### Statistical Analysis

Statistical analysis was performed using GraphPad Prism v.8.0 software. The results were analyzed using one‐way or two‐way ANOVA or Tukey's post hoc test for multiple comparisons. *P* values were determined using the log‐rank test. The results are expressed as the mean ± SD. *P* values less than 0.05 were considered as statistical significance.

## Conflict of Interest

The authors declare no conflict of interest.

## Author Contributions

Q.Z. and A.Q. contributed equally to this work. Q.Z., A.Q., Y.H., and E.Y.X. performed most of the experiments, analyzed the data, and wrote the manuscript. D.K.P.N., Q.L., and D.L. directed the research and reviewed and edited the manuscript. L.W. and G.Y. performed some of the in vitro and in vivo experiments. Y.S. provided valuable comments, analyzed the data, and edited the manuscript. All authors helped to improve the manuscript.

## Supporting information



Supporting Information

## Data Availability

The data that support the findings of this study are available in the supplementary material of this article.
