# Supplementary material for: Fabrication of Polydopamine‐Coated High‐Entropy MXene Nanosheets for Targeted Photothermal Anticancer Therapy
Source: Adv Sci (Weinh). 2024 Dec 24;12(7):2410537. doi: 10.1002/advs.202410537 (PMC11831489; doi:10.1002/advs.202410537)
Supplement: Supplementary file 1 — Supporting Information [file ADVS-12-2410537-s001.pdf]

ADVANCED SCIENCE
Open Access

Supporting Information

for *Adv. Sci.*, DOI 10.1002/advs.202410537

Fabrication of Polydopamine-Coated High-Entropy MXene Nanosheets for Targeted Photothermal Anticancer Therapy

Qingshuang Zou, Ailin Qiu, Yan He, Evelyn Y. Xue, Lujie Wang, Gun Yang, Yao Shen, Dixian Luo, Quan Liu* and Dennis K. P. Ng**

Supporting Information

Fabrication of Polydopamine-Coated High-Entropy MXene Nanosheets for Targeted Photothermal Anticancer Therapy

Qingshuang Zou,† Ailin Qiu,† Yan He, Evelyn Y. Xue, Lujie Wang, Gun Yang, Yao Shen, Dixian Luo, Quan Liu,* and Dennis K. P. Ng**

Q. Zou, E. Y. Xue, D. K. P. Ng

Department of Chemistry, The Chinese University of Hong Kong, Shatin, N.T., Hong Kong, 999077, China

E-mail: dkpn@cuhk.edu.hk

A. Qiu, Y. He, L. Wang, G. Yang, Y. Shen, D. Luo, Q. Liu

Department of Laboratory Medicine, Huazhong University of Science and Technology Union Shenzhen Hospital (Nanshan Hospital), Shenzhen University, Shenzhen 518052, China

E-mail: luo_dixian@email.szu.edu.cn

liu_quan@ email.szu.edu.cn

A. Qiu, Y. He, L. Wang, G. Yang

Institute of Pharmacy and Pharmacology, School of Pharmaceutical Science, Hengyang Medical School, University of South China, Hengyang, Hunan 421001, China

E. Y. Xue

Department of Biomedical Engineering, The Chinese University of Hong Kong, Shatin, N.T., Hong Kong, 999077, China

[†] These authors contributed equally to this work.

Contents

Figure S1 Preparation of HE-M nanosheets with a suitable size. Part of the figure was created using the icons in BioRender.com.

Figure S2 Preparation of HE-M@PDA-Pc-QRH. Part of the figure was created using the icons in BioRender.com.

Figure S3 Change in electronic absorption spectrum of DPBF (initial concentration = 90 μM) in deionized water with 10% (v/v) DMSO in the presence of A) HE-M@PDA-Pc-QRH, B) HE-M, or C) free Pc upon irradiation (λ_{ex} > 610 nm) over a period of time. The concentration of the nanosheets or Pc was fixed at 80 μg mL^{-1} or 2 μM. D) The results for neat water are also included for comparison.

Figure S4 Temperature rise curves for HE-M, HE-M@PDA, HE-M@PDA-Pc, and HE-M@PDA-Pc-QRH dispersed in deionized water (100 μg mL^{-1}) upon irradiation with an 808 nm laser at 1.5 W cm^{-2} for a period of 360 s.

Figure S5 Temperature rise curves for Ti_3C_2, Nb_2C, black phosphorus, and HE-M dispersed in deionized water (100 μg mL^{-1}) upon irradiation with an 808 nm laser at 1.5 W cm^{-2} for a period of 360 s.

Figure S6 A) Natural cooling curve of HE-M after reaching the maximum temperature upon laser irradiation at 808 nm (1.5 W cm^{-2}). B) Plot of time t against the negative natural logarithm of function θ, where $\theta = \frac{T - T\text{surr}}{T\text{max} - T\text{surr}}$, during the cooling period according to the equation $t = -\tau_s \ln\theta$. The solid line represents the line of best fit, from which the value of τ_s was determined to be 133 s.

Figure S7 Confocal images of 4T1 cells after incubation with different concentrations of HE-M@PDA-Pc-QRH for different periods of time.

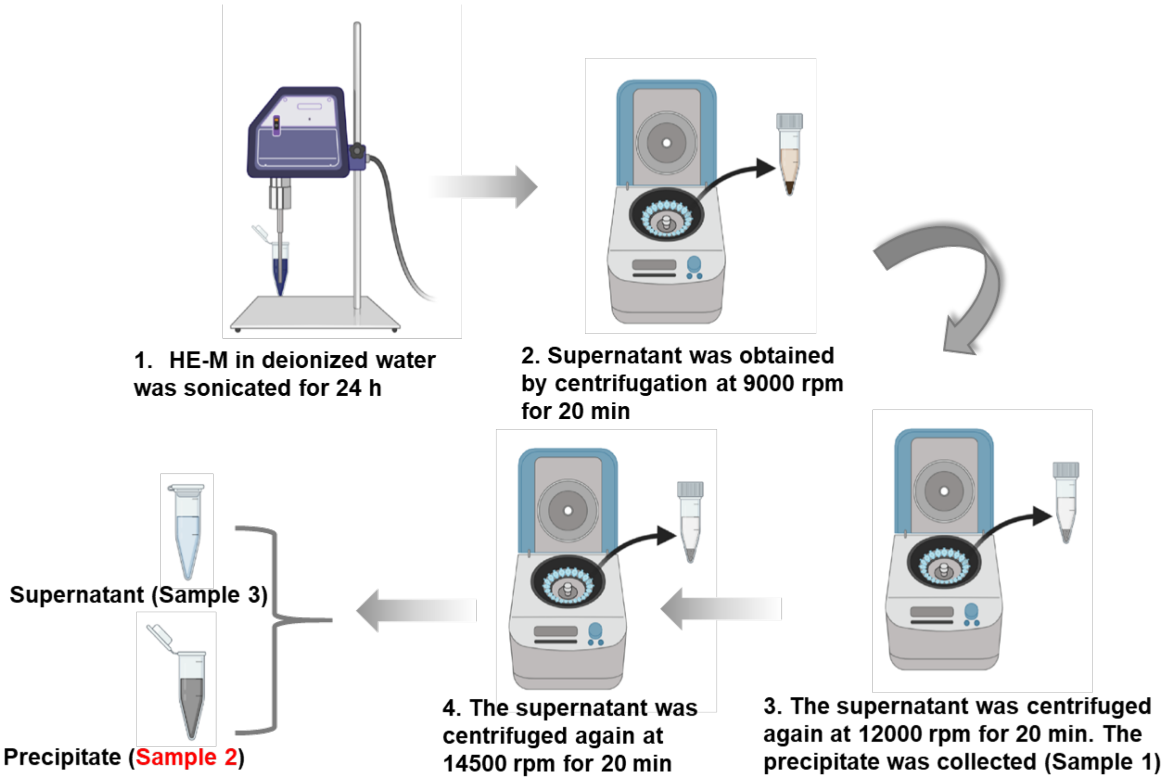

Sample	Ultrasonic Power and Time	Rotational Speed (rpm)	Time	Size (DLS Results)
Sample 1	0.46 kW/h 24 h	9000-12000	20 min	123.7± 9.1 nm
**Sample 2**		**12000-14500**		**94.8 ± 3.6 nm**
Sample 3		≥14500		87.3 ± 5.5 nm

Figure S1. Preparation of HE-M nanosheets with a suitable size. Part of the figure was created using the icons in BioRender.com.

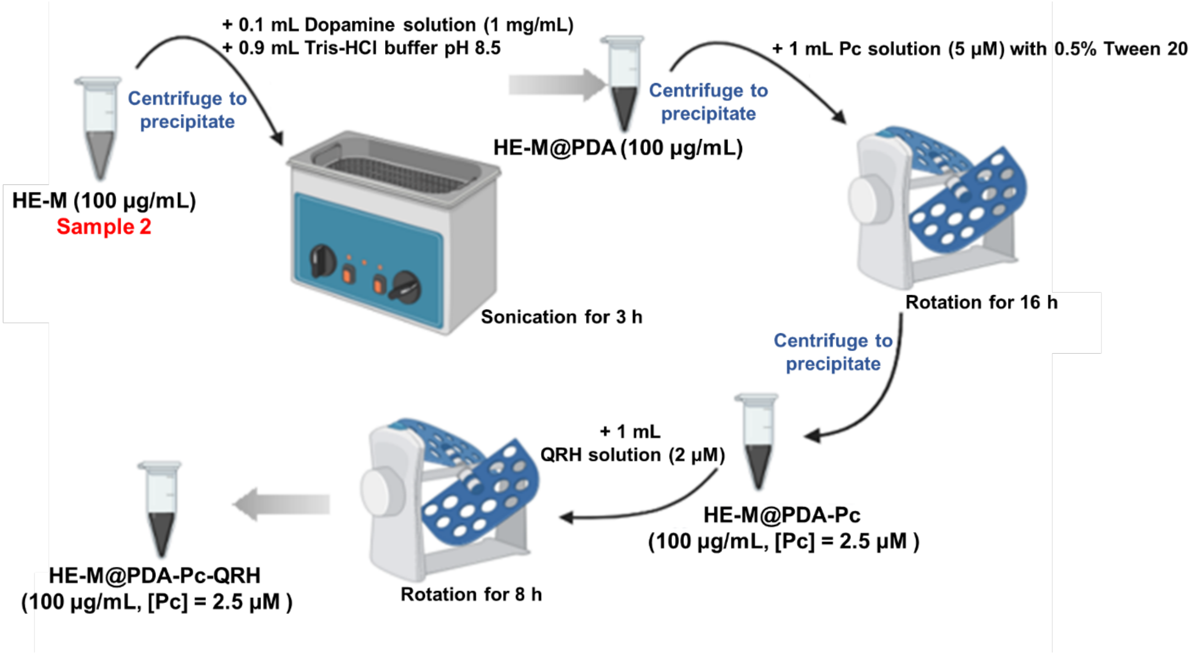

Figure S2. Preparation of HE-M@PDA-Pc-QRH. Part of the figure was created using the icons in BioRender.com.

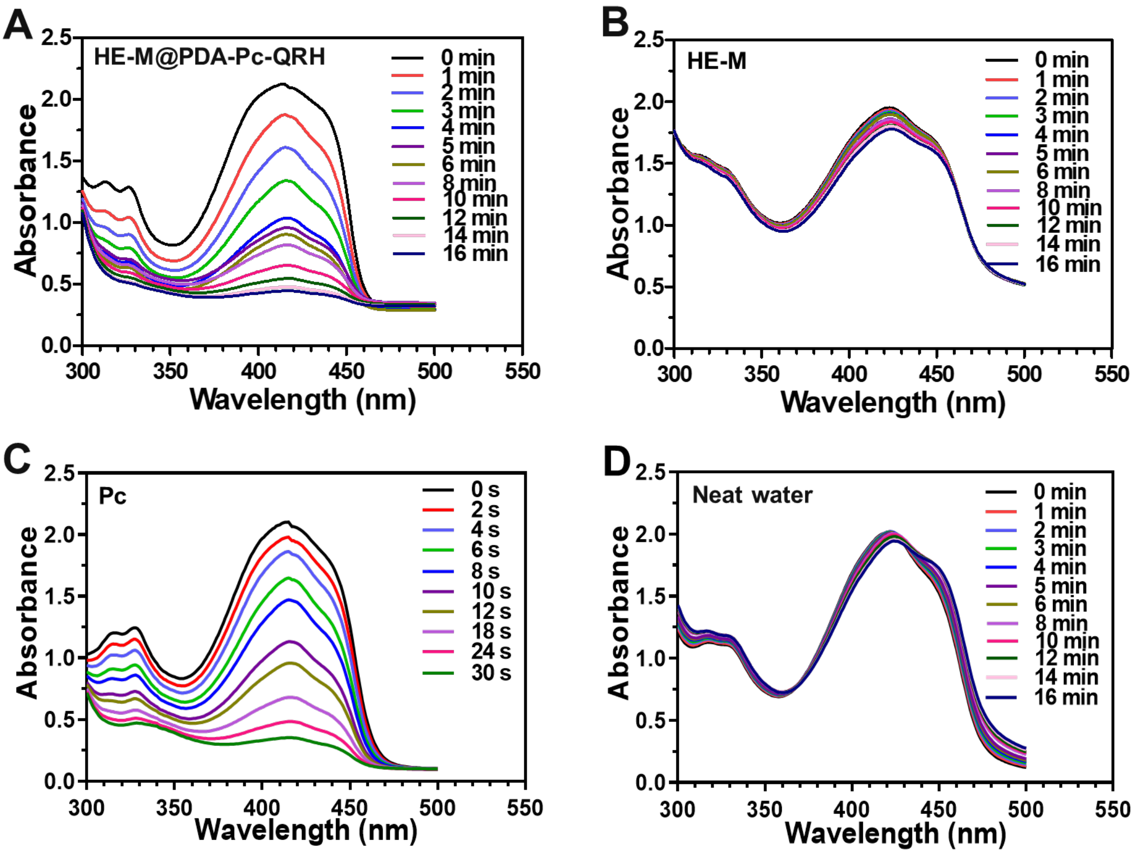

Figure S3. Change in electronic absorption spectrum of DPBF (initial concentration = 90 μM) in deionized water with 10% (v/v) DMSO in the presence of A) HE-M@PDA-Pc-QRH, B) HE-M, or C) free Pc upon irradiation (λ_{ex} > 610 nm) over a period of time. The concentration of the nanosheets or Pc was fixed at 80 μg mL^{-1} or 2 μM. D) The results for neat water are also included for comparison.

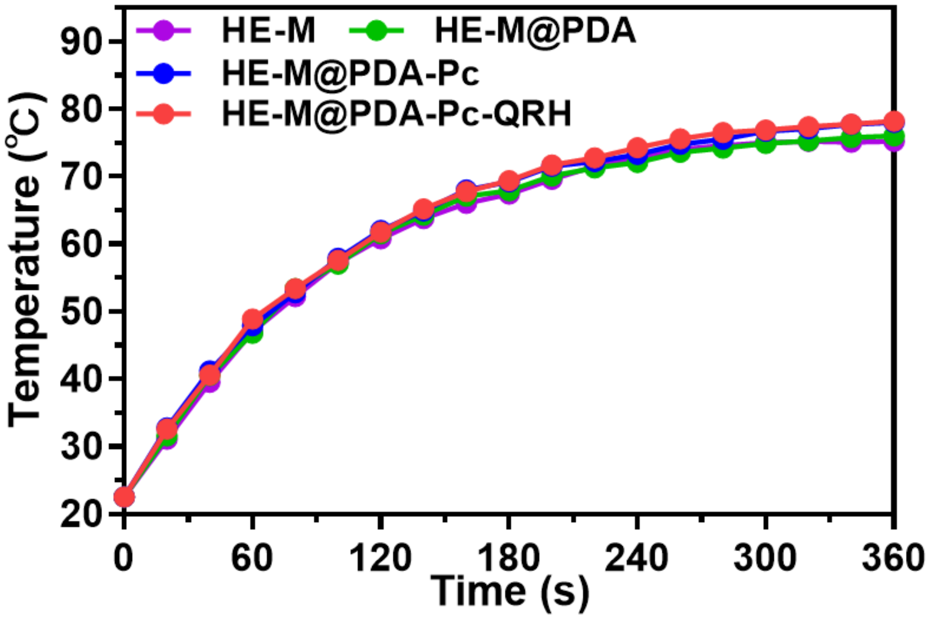

Figure S4. Temperature rise curves for HE-M, HE-M@PDA, HE-M@PDA-Pc, and HE-M@PDA-Pc-QRH dispersed in deionized water (100 μg mL^{-1}) upon irradiation with an 808 nm laser at 1.5 W cm^{-2} for a period of 360 s.

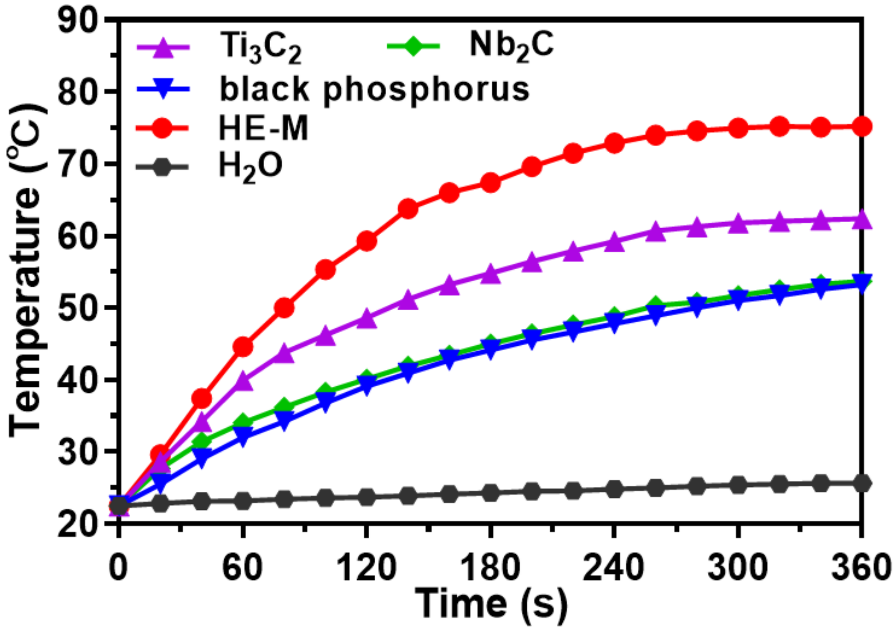

Figure S5. Temperature rise curves for Ti_3C_2, Nb_2C, black phosphorus, and HE-M dispersed in deionized water (100 μg mL^{-1}) upon irradiation with an 808 nm laser at 1.5 W cm^{-2} for a period of 360 s.

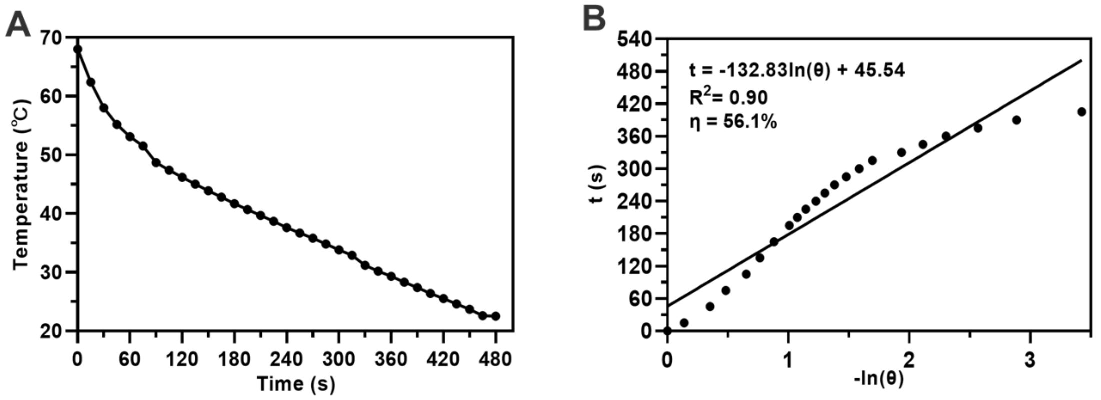

Figure S6. A) Natural cooling curve of HE-M after reaching the maximum temperature upon laser irradiation at 808 nm (1.5 W cm^{-2}). B) Plot of time t against the negative natural logarithm of function θ, where $\theta = \frac{T - T\text{surr}}{T\text{max} - T\text{surr}}$, during the cooling period according to the equation t = -τ_s lnθ. The solid line represents the line of best fit, from which the value of τ_s was determined to be 133 s.

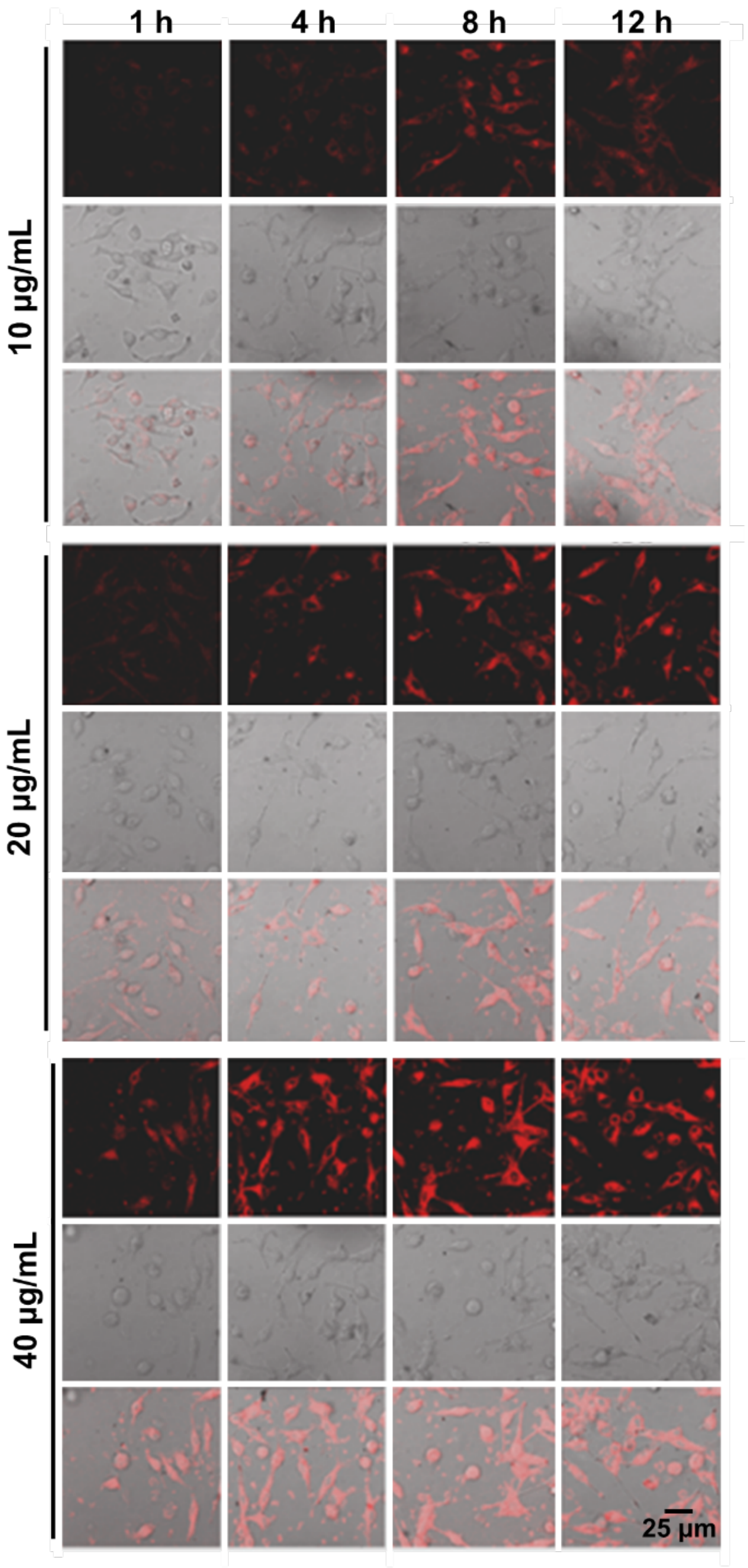

Figure S7. Confocal images of 4T1 cells after incubation with different concentrations of HE-M@PDA-Pc-QRH for different periods of time.
